# Awareness of hypertension and depressive symptoms: a cross-sectional study in a primary care population

**DOI:** 10.1080/02813432.2018.1499588

**Published:** 2018-08-23

**Authors:** Ansa Talvikki Rantanen, Jyrki Jaakko Antero Korkeila, Eliisa Susanna Löyttyniemi, Ulla Kirsti Maria Saxén, Päivi Elina Korhonen

**Affiliations:** a Department of General Practice, Turku University and Turku University Hospital, Turku, Finland;; b Salo Health Center, Salo, Finland;; c Department of Psychiatry, Turku University and Turku University Hospital, Turku, Finland;; d Department of Psychiatry, Hospital District of Satakunta, Pori, Finland;; e Department of Biostatistics, Turku University and Turku University Hospital, Turku, Finland;; f Central Satakunta Health Federation of Municipalities, Harjavalta, Finland

**Keywords:** Beck’s depression inventory, cardiovascular disease, health behavior, hypertension awareness, mental disorders, risk

## Abstract

**Objective:** To investigate the association of hypertension awareness and depressive symptoms, and to analyse factors predisposing aware hypertensives to depressive symptoms.

**Design:** Cross-sectional study in a primary care population.

**Setting:** Cardiovascular risk factor survey in two semi-rural towns in Finland.

**Subjects:** Two thousand six hundred seventy-six middle-aged risk persons without an established cardiovascular or renal disease or type 2 diabetes.

**Main outcome measures:** Depressive symptoms, previous and new diagnosis of hypertension.

**Results:** Hypertension was diagnosed in 47.9% of the subjects, of whom 34.5% (442/1 282) had previously undetected hypertension. Depressive symptoms were reported by 14% of the subjects previously aware of their hypertension, and by 9% of both unaware hypertensives and normotensive subjects. In the logistic regression analysis, both the normotensive (OR 0.62, 95% CI 0.45–0.86) (*p* = 0.0038) and the unaware hypertensive subjects (OR 0.54, 95% CI 0.35–0.84) (*p* = 0.0067) had lower risk for depressive symptoms than the previously diagnosed hypertensives. Among these aware hypertensives, female gender (OR 3.61, 95% CI 2.06–6.32), harmful alcohol use (OR 2.55, 95% CI 1.40–4.64) and obesity (OR 2.50, 95% CI 1.01–6.21) predicted depressive symptoms. Non-smoking (OR 0.57, 95% Cl 0.33–0.99) and moderate leisure-time physical activity compared to low (OR 0.53, 95% CI 0.33–0.84) seemed to buffer against depressive symptoms.

**Conclusion:** Depressive symptoms are common in hypertensive persons even without comorbidities, if the person is already aware of his/her hypertension. Many modifiable, lifestyle associated factors may contribute to the association of hypertension and depressive symptoms.

**Key Points**
- Hypertension and depressive symptoms are known to form a toxic combination contributing even to all-cause mortality.
- Comorbidities or the labelling effect of the diagnosis of hypertension can confound their association.
- Our study shows that depressive symptoms are common in hypertensive persons even without comorbidities, if the person is already aware of his/her hypertension.
- Many modifiable, lifestyle-associated factors may contribute to the association of hypertension and depressive symptoms.
- When treating hypertensive patients, consideration of depressive symptoms is important in order to promote favorable lifestyle and control of hypertension.

## Introduction

Hypertension affects every third adult worldwide [1] and it is the most important risk factor for disability-adjusted life-years [2]. In primary care setting, approximately 10% of patients suffer from depression and another 10% have depressive symptoms [3]. Based on a large Swedish population registry, Sandström et al. recently showed that individuals with the diagnosis of hypertension are also more commonly diagnosed with depression than individuals without hypertension [4]. The coexistence of self-reported hypertension and depressive symptoms was associated with a 15% higher relative hazard for all-cause mortality than hypertension without depressive symptoms even after adjustment for lifestyle factors and comorbid diseases; as reported in the US National Health and Nutrition Epidemiologic Follow-up Study (NHANES) [5]. Thus, when present simultaneously, hypertension and depressive symptoms form a toxic combination highlighting the importance of the recognition of depressive symptoms in hypertensive subjects.

However, comorbidities such as cardiovascular disease (CVD), diabetes, and obesity may have confounding effects on the association between hypertension and mental distress. It has also been suggested that having the diagnosis of hypertension may partly explain the higher level of psychological distress in patients treated for hypertension [6,7]. This awareness of hypertension may have a labelling effect causing mental distress, as suggested by Hamer et al. [6], or it may be the result of increased healthcare utilization of depressed persons as proposed by Michal et al. [7], who also reported that unawareness of hypertension was inversely associated with depression. In these studies, a substantial proportion of patients had CVD or diabetes, and blood pressure was measured only on one occasion. We had the opportunity to investigate the association between hypertension awareness and depressive symptoms in subjects who had no manifested CVD, renal disease or diabetes. The diagnosis of previously unknown hypertension was confirmed with home blood pressure measurements and thus, we could exclude subjects with white coat hypertension. We hypothesized that having a diagnosis of hypertension, rather than elevated blood pressure *per se*, is associated with depressive symptoms. Our secondary aim was to investigate factors predisposing subjects with known hypertension to depressive symptoms.

## Methods

### Study population

The study population was drawn from the Harmonica Project (Harjavalta Risk Monitoring for Cardiovascular Disease), a population survey carried out in Harjavalta and Kokemäki (7,646 and 8,217 inhabitants on December 31, 2007, respectively) from August 2005 to September 2007. All home-dwelling inhabitants aged 45–70 years (*n* = 6,013) were invited, and mailed a cardiovascular risk factor survey, a tape for waist circumference measurement, and a type 2 diabetes risk assessment form (FINDRISC, Finnish Diabetes Risk Score, available from www.diabetes.fi/english). The participation rate was 74% (4,450/6,013). Those having at least one cardiovascular risk factor (*n* = 3,072) were invited for an enrolment examination performed by a trained study nurse. Risk factors taken into account were the latest blood pressure measurement ≥140/90 mmHg, use of antihypertensive medication, history of gestational diabetes or hypertension, family history of premature CVD, waist circumference at the level of the navel ≥80 cm in women and ≥94 cm in men in Harjavalta, and FINDRISC-score ≥12 points in Harjavalta or ≥15 points in Kokemäki. Subjects with known CVD, renal disease or diabetes were excluded.

### Measurements

Blood pressure (BP) was measured by a trained nurse with a mercury sphygmomanometer with subjects in a sitting posture, after resting for at least 5 minutes with the cuff placed on the arm. If the mean of two readings of systolic BP was ≥140 mmHg or diastolic BP ≥90 mmHg and the subjects had no antihypertensive medication, they were taught to use an automatic validated BP monitor (Omron^®^ M4-1, Japan) which was lent to them for home BP monitoring. The participants whose arm circumference was ≥32 cm used a larger cuff. The subjects were advised to perform duplicate BP measurements in a seated position after five minutes of rest in the morning and evening for one week. The recorded measurements except those from the first day were used to calculate the mean home BP. The subjects were classified according to their hypertension status to three categories:

1. aware hypertensives (subjects with antihypertensive medication)
2. unaware hypertensives (no antihypertensive medication, the mean of home BP monitoring ≥135 mmHg for systolic or ≥85 mmHg for diastolic BP)
3. normotensives

Height and weight were measured with subjects in a standing position without shoes and outer garments, and waist circumference at the level midway between the lower rib margin and iliac crest. Body mass index (BMI) was calculated as weight (kg) divided by the square of height (m^2^). Classification of BMI was as follows: normal weight BMI <25.0 kg/m^2^, overweight BMI 25.0–29.9 kg/m^2^, obese BMI 30.0–34.9 kg/m^2^, very obese BMI ≥35.0 kg/m^2^.

Laboratory tests were determined in blood samples obtained after at least 12 hours of fasting. Glucose values were measured from capillary blood with HemoCue Glucose 201+ system (Angelholm, Sweden). Plasma total cholesterol was measured enzymatically (Olympus^®^ AU640, Japan).

### Questionnaires

The study subjects completed self-administrated questionnaires at the clinic before the enrolment examination was performed: upper secondary school (accomplished/not), cohabiting (yes/no), current smoking (yes/no), alcohol consumption (Alcohol Use Disorders Identification Test, AUDIT [8]), leisure-time physical activity (LTPA) level, and Beck’s Depression Inventory (BDI) [9]. AUDIT score ≥8 was regarded as the cut-off for harmful alcohol use [8]. LTPA was classified as follows: high: LTPA for at least 30 minutes at a time for six or more times a week; moderate: LTPA for at least 30 minutes at a time for four to five times a week; low: LTPA for at least 30 minutes at a time for maximum of three times a week. The presence of clinically significant depressive symptoms was defined as a BDI score ≥13 [10], indicating at least mild depression.

### Ethical approval

The ethics committee of Satakunta hospital district reviewed and approved the study protocol and consent forms. All participants provided written informed consent for the project and subsequent medical research.

### Statistical analyses

Descriptive statistics are shown as the number of subjects and proportions for categorical variables. For normally distributed variables, means with standard deviations (*SD*) are presented, and median with interquartile range (IQR) otherwise. Characteristics of normotensive, unware hypertensive and aware hypertensive subjects were compared with one-way analysis of variance, Kruskal–Wallis test or chi-square test. Predictors for depressive symptoms (BDI ≥13) were modelled with logistic regression with the following independent variables: hypertension status, age, gender, education, cohabiting, smoking, alcohol use, LTPA, BMI as categorised. In addition, similar model was executed to subjects using antihypertensive medication including medication (beta-blocker, calcium antagonist, angiotensin-converting enzyme inhibitor or angiotensin receptor antagonist, diuretic). Odds ratios (OR) and their 95% confidence intervals were calculated from this logistic regression model. All statistical tests were performed as 2-sided, with a significance level set at 0.05. The analyses were performed using SAS^®^ System, version 9.4 for Windows (SAS Institute Inc., Cary, NC, USA) and SPSS Statistics (IBM SPSS Statistics for Windows, Version 22.0. Armonk, NY, USA).

## Results

### Characteristics of the study population

We evaluated 2,676 cardiovascular risk persons with a mean age of 58 (*SD* 7) years (55.7% women), who had no previously known CVD, diabetes, or renal disease. Characteristics and health behaviours of the subjects are presented in Table 1. Hypertension was diagnosed in 1 282 (47.9%) of the subjects, of whom 442 (34.5%) had not previously been detected as having hypertension and were thus unaware of the disease. Normotensive subjects were slightly younger, more often female, more educated, and less frequently at-risk users of alcohol than the hypertensive subjects. The prevalence of obesity (BMI ≥30.0 kg/m^2^) was 49.9% (419/840) among aware hypertensive, 29.9% (132/442) among unaware hypertensives, and 21.5% (300/1,394) among normotensive subjects (*p* < 0.001). The mean of BMI was 29.5 kg/m^2^ in female subjects and 28.8 kg/m^2^ in males.

**Table 1.** Characteristics of the subjects according to hypertension status.

	Normotensives *n* = 1394	Unaware hypertensives *n* = 442	Aware hypertensives*n* = 840	*p*-value
Age, mean, years (*SD*)	57 (7)	59 (7)	60 (7)	<0.001
Females, *n* (%)	814 (58.4)	218 (49.3)	458 (54.5)	0.003
Upper secondary school, *n* (%)	239 (17.8)	54 (12.7)	91 (11.3)	<0.001
Cohabiting, *n* (%)	1030 (76.6)	352 (82.6)	630 (77.7)	0.031
Depressive symptoms (BDI ≥13), *n* (%)	129 (9.3)	38 (8.7)	116 (14.1)	<0.001
BDI-score, median (IQR)	4.0 (2.0–8.0)	4.0 (2.0–7.0)	5.0 (3.0–9.0)	<0.001
Current smoking, *n* (%)	258 (18.5)	80 (18.1)	131 (15.6)	0.202
AUDIT-score ≥8, *n* (%)	251 (18.7)	111 (25.8)	162 (20.1)	0.006
LTPA, *n* (%)				0.219
Low	445 (34.0)	162 (37.9)	312 (38.9)	
Moderate	683 (51.1)	207 (48.4)	383 (47.7)	
High	199 (14.9)	59 (13.8)	108 (13.4)	
Systolic blood pressure, mmHg, mean (*SD*)	134 (17)	157 (16)	146 (17)	<0.001
Diastolic blood pressure, mmHg, mean (*SD*)	81 (9)	91 (9)	86 (10)	<0.001
Total cholesterol, mmol/l, mean (*SD*)	5.4 (1.0)	5.4 (0.9)	5.1 (1.0)	<0.001
Fasting glucose, mmol/l, mean (*SD*)	5.5 (1.1)	5.6 (1.0)	5.8 (1.3)	<0.001
Body mass index, kg/m^2^, mean (*SD*)	28.2 (4.7)	28.5 (4.6)	30.7 (5.4)	<0.001
Body mass index category, *n* (%)				<0.001
<25 kg/m^2^	207 (20.7)	81 (19.5)	87 (10.6)	
25.0–29.9 kg/m^2^	491 (49.2)	203 (48.8)	311 (38.1)	
30.0–34.9 kg/m^2^	228 (22.8)	101 (24.3)	277 (33.9)	
≥35.0 kg/m^2^	72 (7.2)	31 (7.5)	142 (17.4)	
Current medication, *n* (%)				
Antidepressant	28 (2.0)	17 (3.8)	45 (5.4)	<0.001
Beta-blocker	0	0	433 (51.5)	<0.001
Calcium antagonist	0	0	233 (26.5)	<0.001
ACE inhibitor or ATR antagonist	3 (0.2)	0	473 (56.3)	<0.001
Diuretic	2 (0.1)	0	231 (27.5)	<0.001
Statin	61 (4.4)	25 (5.7)	184 (21.9)	<0.001

BDI: Beck´s depression inventory; AUDIT: Alcohol Use Disorders Identification Test; LTPA: leisure-time physical activity; ACE: angiotensin-converting enzyme; ATR: angiotensin receptor.

### Association of hypertension status and depressive symptoms (BDI ≥13)

Depressive symptoms were reported by 14.1% (116/822) of the aware hypertensives, by 8.7% (38/439) of the subjects unaware of their hypertension and by 9.3% (129/1,382) of the normotensive subjects. In the logistic regression analysis, the difference in depressive symptoms between hypertension status categories remained significant (*p* = 0.0030) despite common risk factors. When compared to aware hypertensives, both the normotensive (OR 0.62, 95% CI 0.45–0.86) (*p* = 0.0038) and the unaware hypertensive subjects (OR 0.54, 95% CI 0.35–0.84) (*p* = 0.0067) had a lower risk for depressive symptoms.

### Predictors of depressive symptoms among aware hypertensives

Table 2 shows the predictors of depressive symptoms among the aware hypertensives. The strongest predictor was female gender (OR 3.61, 95% CI 2.06–6.32), followed by harmful alcohol use (OR 2.55, 95% CI 1.40–4.64) and obesity (OR 2.50, 95% CI 1.01–6.21). Non-smoking (OR 0.57, 95% Cl 0.33–0.99) and moderate LTPA compared to low (OR 0.53, 95% CI 0.33–0.84) seemed to buffer against depressive symptoms. These factors were independently associated with depressive symptoms also among the whole study population.

**Table 2.** Predictors of depressive symptoms defined as Beck’s Depression Inventory score ≥13 among aware hypertensives.

Variables	OR (95% Cl)	*p*-value
Age	1.01 (0.98–1.05)	0.59
Female gender	3.61 (2.06–6.32)	<0.001
Upper secondary school	1.24 (0.66–2.33)	0.506
Cohabiting	1.11 (0.67–1.85)	0.677
Non-smoking	0.57 (0.33–0.99)	0.048
Harmful alcohol use	2.55 (1.40–4.64)	0.002
Leisure-time physical activity		0.026
Low	1.00 (reference)	
Moderate	0.53 (0.33–0.84)	
High	0.79 (0.39–1.59)	
Body mass index category		0.033
<25.0 kg/m^2^	1.00 (reference)	
25.0–29.9 kg/m^2^	1.51 (0.59–3.87)	
≥30.0 kg/m^2^	2.50 (1.01–6.21)	
Current medication		
Beta-blocker	1.63 (1.00–2.68)	0.052
Calcium antagonist	0.72 (0.42–1.23)	0.228
ACE inhibitor or ATR antagonist	1.22 (0.74–2.01)	0.432
Diuretic	0.80 (0.48–1.32)	0.377

ACE: angiotensin-converting enzyme; ATR: angiotensin receptor.

## Discussion

In this study, we found that clinically significant depressive symptoms were significantly and independently associated with elevated blood pressure, if the subject was already aware of his/her hypertension. Secondly, in our sample clinically significant depressive symptoms were associated with female gender, harmful alcohol use and obesity. Non-smoking and moderate LTPA seemed to buffer against depressive symptoms.

The main strengths of our study are a representative sample of people typically treated in primary care, exclusion of subjects with known cardiovascular or renal disease and diabetes diminishing confounding by comorbidities and the use of home blood pressure measurements excluding confounding by white coat hypertension. Our results suggest that the association of hypertension and depressive symptoms may be modified by several factors independently associated with depressive symptoms. These have not always been taken into account in medical studies, probably contributing to the mixed results of previous research. The present study has also limitations. Because of the cross-sectional study design, we cannot determine any causality between hypertension and depressive symptoms. Although we were able to take into account many factors influencing both depressive symptoms and hypertension, it is possible that factors such as other causes of mental distress and number of health care visits confound the found association. Our study population only included subjects considered to be at risk of a cardiovascular disease, which must be considered when applying our results to the general population. As only subjects with elevated blood pressure were instructed to perform home blood pressure measurements, we cannot exclude the possibility of masked hypertension.

In keeping with our findings, Hamer et al. observed an elevated risk of psychological distress in patients treated for hypertension, but not in unaware hypertensives, compared to normotensive subjects in a large population-based study conducted in England and Scotland [6]. Also Michal et al. [7] found controlled, but not uncontrolled, treated hypertension being associated with increased risk for depression in a German population-based cohort study. In contrast to Hamer’s and our findings, they also found the unawareness of hypertension having a buffering effect against depressive symptoms. The investigators suggested that these findings are mainly explained by increased healthcare utilization of depressed persons rather than a labelling effect [7]. The reason for these somewhat mixed results can be the misclassification of the hypertension status of the subjects and confounding by comorbidity. In the above-mentioned studies, hypertension status was determined by a single measurement and thus the possible misclassification of subjects with white coat hypertension to unaware hypertensives may influence the results. We cannot discount the possibility that depressed individuals use more healthcare services, which enhances the chance of diagnostic recognition of hypertension as suggested by Michal et al. [7]. In Finland, anxiety has been associated with increased utilization of health care services [11]. Only 5% of the previously diagnosed hypertensives in our study population used antidepressant medication suggesting a low level of clinically significant depression or underdiagnosed depression among medicated hypertensives.

In our sample, the total prevalence of depressive symptoms among aware and unaware hypertensive subjects was 22.8%. In a meta-analysis of 10,194 hypertensive subjects in 27 studies using self-rating scales Li et al. [12] found even higher prevalence of depression (29.8%). One study had used BDI, and, with a cut-off point of 16, the prevalence of depression was 26.3% in a sample of hypertensive patients. According to Li et al., self-rating scales as measures may introduce considerable heterogeneity into the results [12]. The difference in the prevalence of depression between our study and the meta-analysis is likely due to different methods used.

The majority of hypertensive patients attending a primary care physician´s office have previously been diagnosed and already been prescribed an antihypertensive medication. Considering the deleterious effects of concomitant hypertension and depression, it is important to recognise depressive symptoms in hypertensive subjects. Our results suggest that female gender, harmful alcohol use, obesity, smoking, and low level of LTPA are factors predisposing hypertensive patients to depression. Depression and depressive symptoms affect a person’s motivation and ability to lead a healthy life. On the other hand, physical activity and other components of a healthy lifestyle promote psychological well-being. Not surprisingly, an unhealthy lifestyle including low physical activity and alcohol intake has been associated with depression [13,14], which is plausible in a bi-directional way. Alcohol use disorder and depressive disorder have been found to double the risk for the other disorder [15]. In addition, obesity is linked to unhealthy lifestyle. A recent meta-analysis [16] provides evidence that depression and obesity are associated bi-directionally: there is a 37% increase in the risk of being obese in depressed adults, and obese adults have an 18% increase in the risk of being depressed. One has to keep in mind, that these lifestyle-connected factors associated with depressive symptoms are also risk factors for hypertension. Hence, contributing to these modifiable risk factors may attenuate the effect of the toxic combination of depressive symptoms and hypertension.

In conclusion, depressive symptoms are common in hypertensive persons even without comorbidities, if the person is already aware of his/her hypertension. Thus, discussing a new chronic condition and lifelong medication should happen in a delicate, supporting manner. Many modifiable, lifestyle-associated factors may contribute to the association of hypertension and depressive symptoms. Clinicians should consider depressive symptoms when treating hypertensive subjects and find ways to enhance their psychological well-being in order to promote a favourable lifestyle and control of hypertension. Future research will hopefully clarify whether treatment of the screen-detected depression would also lower blood pressure levels in hypertensive subjects.
